# A cross sectional study of prevalence, risk factors, population attributable fractions and pathology for foot and limb lesions in preweaning piglets on commercial farms in England

**DOI:** 10.1186/1746-6148-5-31

**Published:** 2009-08-24

**Authors:** Amy L KilBride, Claire E Gillman, Pete Ossent, Laura E Green

**Affiliations:** 1 Department of Biological Sciences, University of Warwick, Coventry, CV4 7AL, UK; 2 Institute for Veterinary Pathology, University of Zürich, Winterthurerstrasse 268, CH-8057 Zürich, Switzerland

## Abstract

**Background:**

In a cross sectional study of 88 indoor and outdoor English pig farms, the prevalence of foot and limb lesions in 2843 preweaning piglets aged 1–4 weeks from 304 litters was recorded. The environmental risks for the prevalence of lesions and population attributable fractions were calculated. The risks for lesions in piglets were compared with those for limb and body lesions in their mothers. A small number of piglets with each type of lesion were examined *post mortem *to elucidate the pathology of the clinical lesions observed.

**Results:**

The prevalence of sole bruising, sole erosion, skin abrasion and swollen joints or claws in 2843 piglets was 49.4% (1404), 15.5% (441), 43.6% (1240) and 4.7% (143) respectively. The prevalence of all foot and limb lesions was higher in indoor housed piglets than in outdoor housed piglets. The prevalence of sole bruising (OR 0.3) and skin abrasion (OR 0.6) decreased with each week of age from 1–4 weeks, but there was no significant association between piglet age and the prevalence of sole erosion or swollen joints and claws. There was an increased prevalence of sole bruising (OR 3.0) and swollen joints or claws (OR 3.0) and a decreased prevalence of skin abrasion (OR 0.3, piglets ≤ 1-week old), in piglets housed on slatted floors, compared with those on solid concrete floors with bedding. There was an increased risk of sole erosion associated with piglets housed on partly slatted floors with no bedding (OR 2.4) and partly slatted floors with small amounts of bedding (OR 2.9) compared with piglets housed on solid concrete floors with bedding in all areas of the pen. *Post mortem *examination of feet with lesions indicated that internal pathological changes were frequently more severe than the degree of external damage suggested.

**Conclusion:**

Piglets housed outdoors had a very low prevalence of foot and limb injuries. Indoors, no one floor type was ideal to minimise all piglet foot and limb injuries and the flooring requirements of sows differed from those of piglets.

## Background

Farrowing pen floors made from solid concrete or metal or plastic slats are much harder than the soil surface for which piglets' feet and limbs have evolved. Piglets housed on such floors often develop hairless patches or abrasions on the skin of their limbs [1-5] and bruising or erosion on the soles of their feet [2,3,6,7]. These injuries may become infected if invaded by pathogens, resulting in swollen joints or claws [4,7,8].

The prevalence of skin abrasion, sole bruising, sole erosion and swollen joints or claws on single farms have been estimated to be 80–89% [4,7], 87–100% [6,7], 28% [6] and 6–8% [6,9] respectively. In the only cross sectional study to date, the prevalence of skin abrasion was 36% and the prevalence of sole bruising was 50% in 264 piglets from 13 convenience selected farms in England, the prevalence of other lesions was not recorded [3].

Research to date indicates that skin abrasions are caused by kneeling on rough concrete surfaces [1,3,5]. A small quantity of bedding on the concrete offers little protection because it is easily pushed aside, and may even exacerbate abrasions because shards of sawdust or straw can be pushed into the skin [1,3,5,6]. In contrast, sole bruising is less prevalent on solid concrete floors compared with slatted floors, and the risk of sole bruising decreases as the quantity of bedding on solid floors increases [3].

Bruising and abrasion on piglets' feet and limbs have mechanical causes, however the risks for foot and limb infections are multifactorial and determined by contact with infectious pathogens, a damaged epidermis, the piglet's immune response and treatments administered by the farmer [9]. The pathology associated with infection may be severe. Necrotic pododermatitis, osteomyelitis, arthritis and tenosynovitis were reported in the infected claws of seven lame piglets examined *post mortem *[8]. It is possible that the type of floor and the use of bedding could influence contact between piglets and pathogens [10]. However, on an experimental unit in Canada there was no difference in the prevalence of joint infections in piglets reared on different floor types [11].

Consideration of the environmental needs of piglets cannot be separated from the requirements of the lactating sow. The sow's needs include a comfortable surface for lying, sufficient space and a non slip surface for rising and standing, separation from excreta and a pen that is robust to her size and weight.

In this paper, the prevalence, risks and population attributable fractions for foot and limb lesions in preweaning piglets are presented. The risks are compared with those associated with limb and body lesions in the mothers of these piglets [12]. In addition, the pathology associated with examples of foot and limb lesions in piglets is reported.

## Methods

### Sample size

The data presented in this paper were collected as part of a larger study investigating the impact of commercial pig flooring on pigs of all ages, therefore selection criteria of breeder-to-finisher units with more than 100 breeding sows was applied. Assuming 95% of herds have piglets with foot and limb injuries [3], an approximate population of 1870 (number of herds fitting selection criteria in 2003 in Britain, DEFRA, personal communication), a 95% confidence interval and 5% precision; it was calculated that it would be necessary to sample piglets from 75 farms.

Assuming an approximate study population of 650 000 preweaning piglets on the target farms (DEFRA, 2003 data, personal communication), 50% lesion prevalence, a 95% confidence interval and 5% precision, with an intraclass coefficient of 0.1 at the level of farm and litter [13] it was calculated that a sample size of approximately 3000 preweaning piglets was required to estimate prevalence. To detect a two fold difference in risk between exposed and unexposed piglets with 95% confidence and 80% power, given a 10% prevalence of disease in the unexposed piglets, with an estimated farm and litter intracluster correlation coefficient of 0.1, a sample size of approximately 2700 piglets was required. Sample size calculations were carried out in Win Episcope 2.0.

### Farm selection

A total of 549 breeder-finisher pig farms with more than 100 breeding sows in England and Wales were randomly selected from the Assured British Pig (ABP) database. A total of 101 farmers agreed to take part in the study (18% compliance); 7 of these farms were used to pilot test the recording systems and to train observers. Usable data on preweaning piglets were collected from 89 farms. There was only one farm in the study located in Wales so this farm was excluded from calculations of prevalence and population attributable fractions (n = 88). The Welsh farm, and a further 9 farms that were non-randomly selected for participation (5 from Scotland, recruited by Quality Meat Scotland and 4 from England recruited via their veterinarian), were included in the risk factor analysis giving a total of 98 farms. Because the quantity of missing data varied by outcome and predictor, the number of piglets or litters used in each analysis is reported on each table of results.

### Piglet observations

A comprehensive protocol was written detailing every lesion and score definition. Scoring systems were tested and compared on seven on-farm training days before data collection. On each farm four litters, one each aged 3–7, 8–14, 15–21 and 22–28 days of age were randomly selected using random number tables (counting from the first pen/hut on the left of the entrance). All piglets in the selected litters were examined. All four limbs and feet were examined for foot and limb injuries (Table 1) whist the piglet was restrained by the observer. Eight researchers (all with science or agricultural degrees, experience with pigs and trained for this project) recorded data on the piglets.

**Table 1:** Definitions of foot and limb lesions

**Lesion classification**	**Description**
**Limb lesion^1^**	
Hairless patch	Hair is missing but the epidermis is unbroken and no scab is present.
Skin abrasion	Loss of the outer epidermis resulting in an open wound or a healing wound with a scab
**Foot lesion^1,2^**	
Sole bruising	Congestion and bruising of the solar corium presenting as red or brown pigmentation
Sole erosion	Loss of horny tissue
**Infection**	
Swollen joint or claw	Swelling of the tarsal, carpal, carpophalangeal, digital joint or the claws of the foot

^1^[3]

^2 ^[2]

The size of the hairless patch, skin abrasion, sole bruising or sole erosion was scored on a 0–3 scale with 0 = no visible damage, 1 = damage on <25%, 2 = damage 25–50% and 3 = damage >50% of the surface area of the joint of the limb or the volar surface of the foot. The size of a swollen joint or claw was scored on a 0–3 scale by comparison with the size of the matching unaffected joint or claw with 0 = no visible swelling, 1 = swollen to <25%, 2 = swollen to 25–50%, 3 = swollen to >50% larger than the size of the normal joint/claw.

### Farrowing pen observations

Observers recorded data on the pen or hut environment (Table 2). Indoor farrowing pen floors were divided into three areas to assess their condition; the pen outside the crate, the anterior part of the floor inside the crate, here after referred to as the sow lying area, and the sow dunging area.

**Table 2:** Definitions of variables observed in pens and paddocks

**Variable**	**Definition**
**Pen construction**	
Floor type	Solid, partly slatted or fully slatted
Floor material	Soil, concrete, metal or plastic
**Bedding**	
Bedding location	Outside the crate, sow lying area inside the crate, or sow dunging area inside the crate
Bedding type	Straw, wood shavings or paper
**Floor condition**	
Cleanliness	Wet
- presence or absence	Dry slurry
	Wet slurry
	Spilled food
	Fresh dung
Damage	Sharp edges
- presence or absence	Broken/cracked
	Worn rough surface

### Pathology

Two farms were selected; one with farrowing houses with partly solid concrete/partly slatted cast iron floors and one with farrowing houses with fully slatted plastic floors. Two samples of sole bruising, sole erosion, skin abrasion and swollen joints of each score 0–3 were selected from each farm. Pigs were euthanased and examined *post mortem *by a pathologist (PO). The claws and samples from the limb lesions were preserved in formalin. Relevant tissues were then routinely embedded in paraffin and H and E stained sections were examined histologically. Each lesion was described by the pathologist using gross and histological examination and the severity of the internal lesion was compared with the clinical presentation. The depth of the horn layer on the heel of the feet was measured.

### Data checking and data analysis

Research assistants entered data into Microsoft Access 2003 databases. The databases were checked for errors and outliers and obviously incorrect codes were re-checked against the raw data and impossible values were coded as missing.

Lesion prevalence was calculated separately for each type of lesion. A piglet was defined as affected with a particular lesion if one or more lesions greater than score zero were present on any foot or limb. When piglets had multiple lesions of the same type, the score of the largest lesion was used in analysis. The crude prevalence for each different type of lesion was calculated in the pigs from the ABP farms as follows;



The outcome variable used in the risk factor analysis was the proportion of piglets affected within the litter. The outcome was;



The data had a multilevel structure. That is, litters within the same farm were more likely to be similar (correlated) to each other than litters from different farms. To account for this clustering of litters within farms a 2-level binomial logistic regression model was used with litters (level 1) nested within farms (level 2). MLwiN version 2.01 [14] was used for all multilevel analysis. Models were built to compare indoor and outdoor housed piglets. Separate models were built for the indoor pens to investigate floor construction, bedding use and floor condition controlling for age. The risks associated with skin abrasion in piglets 1 week old or less were investigated separately. Finally partly slatted floors with varying amounts of bedding were compared to investigate the effect of slat material and type of bedding on piglet injury.

Age was included in the models throughout the initial screening of variables for all outcomes and forced into the final models. To check for a linear association with the outcome, continuous variables were tested in the model as a categorical variable and examined for a pattern of increasing or decreasing coefficients. Non linear associations were left as categorical variables. Variables were taken forward for multivariable analysis when significant at p < 0.2 [13]. Where variables were highly correlated the most biologically plausible variable, based on biological knowledge and previous research, was selected for inclusion in the model. Both forward addition and backward elimination were used to identify the variables that had a significant association (p < 0.05) with the outcome [13]. Finally, all variables rejected at the screening stage were retested in the final model to check for residual confounding [15].

The model took the form;



Where p_ij _= is the proportion of the litter affected with a particular lesion, investigated with a logit link function, β_0 _= constant, βx is a vector of fixed effects varying at level 1 (ij) or level 2 (j), i is litters, j is farms and v_j _and u_ij _are the level two and level one residual variance respectively.

Observer identity was forced into each final model to investigate whether it altered the interpretation of the fixed effects. The Hosmer-Lemeshow goodness of fit test [13] was used to investigate the difference between observed values and values predicted by the model. Pearson correlation coefficients were calculated to investigate the association between the ordinal score of lesions within piglets. The population attributable fractions for each lesion were calculated for all floor types that were significantly different from soil from the ABP farms in England using;



Where AF_p _is the population attributable fraction, RD is the risk of a lesion in the exposed group minus the risk in the reference category group, p(E+) is the proportion of piglets on each floor type and p(D+) is the proportion of piglets with the lesion on each floor type [13]. Fractions are converted to percentages for presentation of the results

## Results

### Farm and pen characteristics

A total of 3206 piglets from 338 litters were examined; 288 litters were housed indoors and 50 outdoors. The litter size at the time of examination ranged from 3–16 with a mean of 9.7 (SD 1.9). All piglets kept outdoors were housed in huts set on soil with deep straw bedding on the floor. In the 288 litters housed indoors, 11.9% were kept on solid concrete floors with bedding, 19.9% on part slatted floors with bedding, 17.8% on partly slatted floors with some bedding, 35.0% on partly slatted floors without bedding and 15.0% on fully slatted floors. In the 251 pens with slatted floors, 41.4% had metal slats, 43.4% plastic, 14.3% both metal and plastic and 0.8% had concrete slats. Pens with concrete slats were excluded from further analysis because there were only two such pens. Bedding was present in 50.0% of the 288 indoor pens at the time of observation. This was straw in 58.3% and wood shavings in 35.4% of the pens; the remaining 6.3% of pens were bedded with paper or a combination of beddings.

### Prevalence of foot and limb lesions in 2843 preweaning piglets

The prevalence of sole bruising and sole erosion on the piglets' feet was 48.8% and 15.3% respectively. The prevalence of skin abrasions and hairless patches on the limbs was 43.0% and 61.3% respectively. There were 4.7% of piglets with swollen joints or claws. The prevalence and severity of all lesions was lower in piglets housed outdoors compared with piglets housed indoors (Table 3). None of the outdoor housed piglets had swollen joints or claws and the modal maximum lesion severity for all other lesions was one. In indoor housed piglets the modal maximum lesion severity for sole bruising and erosion was one and for hairless patches, skin abrasions and swellings it was two (Table 3).

**Table 3:** Number and percent of 2843 indoor and outdoor housed piglets from 88 English farms with foot and limb lesions score 0 – 3

	Score^1^	Score 0		Score 1		Score 2		Score 3	
**Lesion**		n	%	n	%	n	%	n	%
**Sole bruising**	Indoor	1042	43.0	807	33.3	452	18.6	123	5.1
	Outdoor	415	99.0	3	0.7	1	0.2	0	0.0
**Sole erosion**	Indoor	2010	82.9	281	11.6	104	4.3	29	1.2
	Outdoor	398	95.0	14	3.3	6	1.4	1	0.2
**Skin abrasion**	Indoor	1218	50.2	424	17.5	523	21.6	259	10.7
	Outdoor	400	95.5	19	4.5	0	0.0	0	0.0
**Hairless patch**	Indoor	769	31.7	502	20.7	707	29.2	446	15.7
	Outdoor	330	78.8	53	12.6	33	7.9	3	0.1
**Swollen joint/claw**	Indoor	2291	94.5	43	1.8	56	2.3	34	1.4

^1 ^0 = no visible damage, 1 = damage on <25%, 2 = damage 25–50% and 3 = damage >50% of the surface area of the joint of the limb, the volar surface of the foot or for swollen joints and claws, swollen by this proportion compared to the size of a normal joint or claw.

The prevalence of lesions varied by limb and foot (Table 4), skin abrasions and hairless patches occurred at the highest prevalence on the fore limb carpal joints and at a lower prevalence on the carpophalangeal joints and on the hind limb tarsal joints. There was a slightly higher prevalence of sole bruising on the fore feet compared with the hind and conversely a higher prevalence of sole erosion on the hind feet compared with the fore. Lesions were equally prevalent on the right and left sides (Table 4).

**Table 4:** Number and percent of 2843 piglets from 88 English farms with foot and limb lesions by location

		**Sole bruising**		**Sole erosion**		**Skin abrasion**		**Hairless patch**		**Swollen joint/claw**	
Limb	Location	n	%	n	%	n	%	n	%	n	%
Fore right	Carpal^1^					1032	36.3	1308	46.0	14	0.5
	Carpoph.^2^					532	18.7	1077	37.9	17	0.6
	Foot	1146	40.3	489	17.2					11	0.4
Fore left	Carpal					873	30.7	1305	45.9	26	0.9
	Carpoph.					517	18.2	1109	39.0	26	0.9
	Foot	1140	40.1	478	16.8					14	0.5
Hind right	Tarsal^3^					287	10.1	574	20.2	28	1.0
	Carpoph.									11	0.4
	Foot	1060	37.3	589	20.7					6	0.2
Hind left	Tarsal					279	9.8	583	20.5	9	0.3
	Carpoph.									14	0.5
	Foot	1035	36.4	577	20.3					14	0.5

^1^carpal joint

^2^carpophalangeal joint

^3 ^tarsal joint

The prevalence of foot and limb lesions in preweaning piglets varied by age, floor type and floor condition (Table 5). The farm level prevalence of skin abrasions, sole bruising, sole erosion and swollen joints or claws was 87.6%, 83.1.5, 68.5% and 56.2% respectively.

**Table 5:** Number and percent of preweaning piglets from 88 English farms with foot and limb lesions by age, floor type and floor condition

	**Sole bruising**		**Sole Erosion**		**Skin abrasion**		**Swollen joint/claw**		
	n	%	n	%	n	%	n	%	Total n
Age									
1-week	551	75.8	97	13.3	415	57.1	28	3.9	727
2-week	465	55.4	130	15.5	431	51.4	41	4.9	839
3-week	240	36.9	117	18.0	212	32.6	32	4.9	651
4-week	130	20.8	91	14.5	167	26.7	32	5.1	626
Floor/bedding									
Solid with bedding	124	37.6	41	12.4	167	50.6	13	3.9	330
Partly slatted with bedding	320	58.3	72	13.1	250	45.5	24	4.4	549
Partly slatted with bedding in some areas	351	72.1	146	30.0	293	60.2	40	8.2	487
Part slatted no bedding	546	56.4	215	22.2	432	44.6	53	5.5	969
Fully slatted	253	61.6	49	11.9	209	50.9	34	8.3	411
Outdoor	4	1.0	21	5.0	19	4.5	0	0.0	419
Worn rough sow lying area									
No	1084	56.3	320	16.6	918	47.7	109	5.7	1925
Yes	270	65.5	86	20.9	257	62.4	27	6.6	412
Worn rough sow dunging area									
No	1278	56.5	381	16.8	1117	49.4	120	5.3	2262
Yes	79	63.7	32	25.8	71	57.3	17	13.7	124
Wet floor in the lying area									
No	1273	58.3	430	19.7	1068	48.9	130	6.0	2184
Yes	303	62.5	84	17.3	261	53.8	32	6.6	485
Bedding type									
Straw	327	33.1	98	9.9	335	33.9	25	2.5	989
Wood shavings	270	63.2	96	22.5	232	54.3	29	6.8	427
Paper	49	89.1	8	14.5	39	70.9	1	1.8	55
Slat material									
Metal	424	53.5	179	22.6	356	44.9	41	5.2	792
Plastic	595	63.3	147	15.6	509	54.1	57	6.1	940
Metal and plastic	209	65.3	42	13.1	162	50.6	19	5.9	320

### Risk factors associated with foot and limb lesions in preweaning piglets

#### Sole erosion

There was a reduced risk of sole erosion associated with piglets housed outdoors compared with piglets housed indoors (OR 0.1, CI 0.1, 0.5). Indoors, there was an increased risk of sole erosion in piglets kept on partly slatted floors with bedding in some areas or no bedding compared with those housed on solid concrete floors with bedding throughout the pen. There was no significant difference in the prevalence of sole erosion in piglets housed on partly slatted floors with bedding or fully slatted floors, compared with those on solid concrete floors with bedding. A wet floor in the sow lying area was associated with a reduced risk of sole erosion compared with a dry floor. There was no significant association between the prevalence of sole erosion and the age of the piglet (Table 6).

**Table 6:** Two level logistic binomial models of the risks associated with foot lesions and swollen joints and claws in preweaning piglets from 98 British farms

**All pens**	**Sole bruise** 286 litters		**Sole erosion** 278 litters		**Swollen joint/claw** 284 litters	
Intercept coefficient	2.2		-2.0		-3.8	
	**OR**	**CI**	**OR**	**CI**	**OR**	**CI**
Age	0.3	0.3, 0.4	1.0	0.9, 1.2	1.1	0.9, 1.3
Floor/bedding						
Solid with bedding						
Partly slatted with bedding	2.2	1.1, 4.6	1.3	0.5, 3.0	1.4	0.6, 3.5
Partly slatted with bedding in some areas	4.2	2.0, 9.0	2.9	1.2, 7.1	2.5	1.1, 6.1
Partly slatted no bedding	2.6	1.3, 5.0	2.4	1.1, 5.5	1.7	0.7, 3.9
Fully slatted	3.0	1.4, 6.5	1.3	0.5, 3.3	3.0	1.2, 7.4
Wet sow lying area						
No						
Yes			0.5	0.3, 0.9		
Worn sow dunging area						
No						
Yes					2.8	1.3, 6.0
Random effects	**Var**.	**SE**	**Var**.	**SE**	**Var**.	**SE**
Farms	0.5	0.2	1.1	0.3	0.3	0.2
Pens	1.0	0.2	0.8	0.2	0.6	0.3
Hosmer-Lemeshow goodness-of-fit	**χ^2^**	**P value**	**χ^2^**	**P value**	**χ^2^**	**P value**
	0.5	0.78	6.2	0.10	3.8	0.20

#### Sole bruising

There was a reduced risk of sole bruising associated with outdoor housed piglets compared with indoor housed piglets (OR 0.005, CI 0.002, 0.01). In indoor housed piglets, the risk of sole bruising decreased with each increasing week of age. There was an increased risk of sole bruising associated with being housed on partly slatted floors with and without bedding and fully slatted floors, compared with solid concrete floors with bedding (Table 6).

#### Swollen joints or claws

There was increased risk of swollen joints or claws in pigs housed on partly slatted floors with some bedding and fully slatted floors, compared with those housed on solid concrete floors with bedding. There was an increased risk of swollen joints and claws when the sow dunging area was rough and worn compared with a smooth floor in the sow dunging area (Table 6). On partly slatted floors with bedding, there was a trend for a reduced risk of joint swelling associated with plastic slats compared with metal (OR 0.4, CI 0.2, 1.1).

#### Skin abrasion

There was a reduced risk of skin abrasion in piglets housed outdoors compared with piglets housed indoors (OR 0.04, CI 0.02, 0.07). In indoor housed piglets the risk of skin abrasion decreased with each week of age from 1–4 weeks. There was no significant difference in the prevalence of skin abrasions in piglets aged 1–4 weeks housed indoors on different floor types (Table 7).

**Table 7:** Two level logistic binomial models of the risks associated skin abrasions in preweaning piglets from 98 British farms

**All pens**	**Skin abrasion** 278 litters		**Skin abrasion <1-wk** 71 litters	
Intercept coefficient	1.5		1.2	
	**OR**	**CI**	**OR**	**CI**
Age	0.6	0.5, 0.7		
Floor/bedding				
Solid with bedding				
Partly slatted with bedding	0.6	0.3, 1.1	0.6	0.2, 2.0
Partly slatted with bedding in some areas	1.0	0.6, 1.8	0.8	0.2, 2.8
Partly slatted no bedding	0.7	0.4, 1.1	0.4	0.1, 1.2
Fully slatted	0.9	0.5, 1.7	0.3	0.0, 0.9
Worn sow lying area				
No				
Yes	1.6	1.1, 2.4	3.0	1.5, 6.0
Random effects	**Var**.	**SE**	**Var**.	**SE**
Farms	0.2	0.1	0.7	0.2
Pens	0.8	0.1		
Hosmer-Lemeshow goodness-of-fit	**χ^2^**	**P value**	**χ^2^**	**P value**
	3.2	0.52	0.4	0.98

There was a trend for a reduced risk of skin abrasion associated with piglets 1-week of age or less housed on partly slatted floors with no bedding and a significantly reduced risk on fully slatted floors, compared with piglets housed on solid concrete floors with bedding. There was an increased risk of skin abrasion in piglets 1-week old or less in pens with a worn rough floor surface in the sow lying area compared with a even floor surface (Table 7).

#### Model fit and observer differences

**Figure 1:**
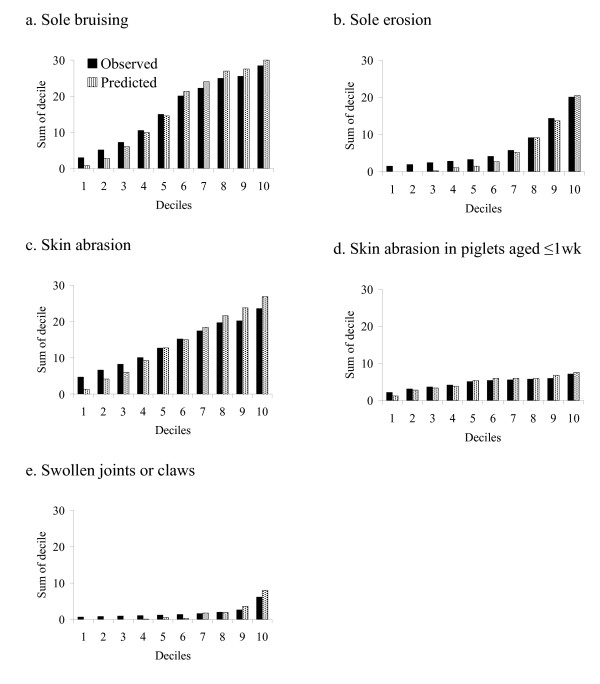
**Graphs a – e observed verses predicted values for foot and limb lesion in preweaning piglets**.

For all models the Hosmer-Lemeshow goodness-of-fit statistic and the graphs indicated that the difference between the observed and predicted values was small (Tables 6 and 7 and Figure 1). Controlling for observer did not alter the interpretation of the fixed effects in any of the models.

#### Associations between limb and foot lesions and slat materials and bedding type

Having accounted for floor type there were no significant associations between slat material (metal or plastic) or bedding type (wood shavings or straw) and the prevalence of any foot and limb lesions in indoor housed piglets (data not shown).

#### Associations between foot and limb lesions

Correlated variables were statistically significant at low values because of the large sample size (Table 8). The strongest statistical correlations were sole bruising positively correlated with skin abrasion and sole erosion, and hairless patches negatively correlated with sole bruising and skin abrasion.

**Table 8:** Pearson correlation coefficients between limb and foot lesions in 3206 indoor and outdoor housed piglets from 98 British farms

	**Sole bruising**	**Sole erosion**	**Skin abrasion**	**Hairless patch**	**Swollen joint/claw**
Sole bruising	1.00				
Sole erosion	0.16*	1.00			
Skin abrasion	0.30*	0.12*	1.00		
Hairless patch	-0.17*	0.13*	-0.20*	1.00	
Swollen joint/claw	0.05*	0.06*	0.12*	0.07*	1.00

* correlated at p < 0.01

#### Pathology

A total of 24 samples of foot and limb lesions were taken for pathological examination from 17 piglets. The median age of the piglets was 7 days (IQR 6, 9). The thickness of the volar heel horn was 1–2 mm.

**Figure 2:**
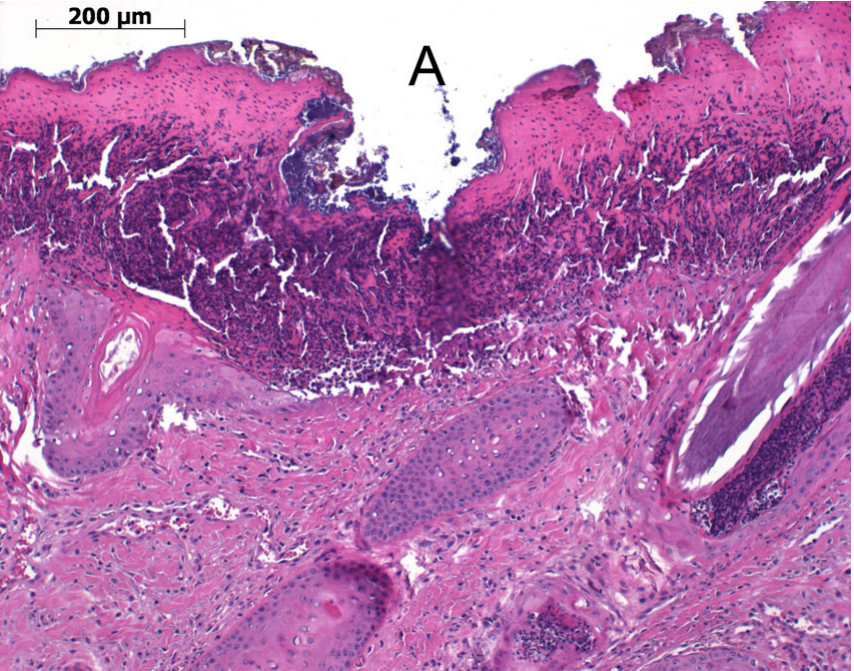
**Histological section of a skin abrasion on the fore limb of a preweaning piglet with inflamation and ulceration of the skin (A)**.

**Figure 3:**
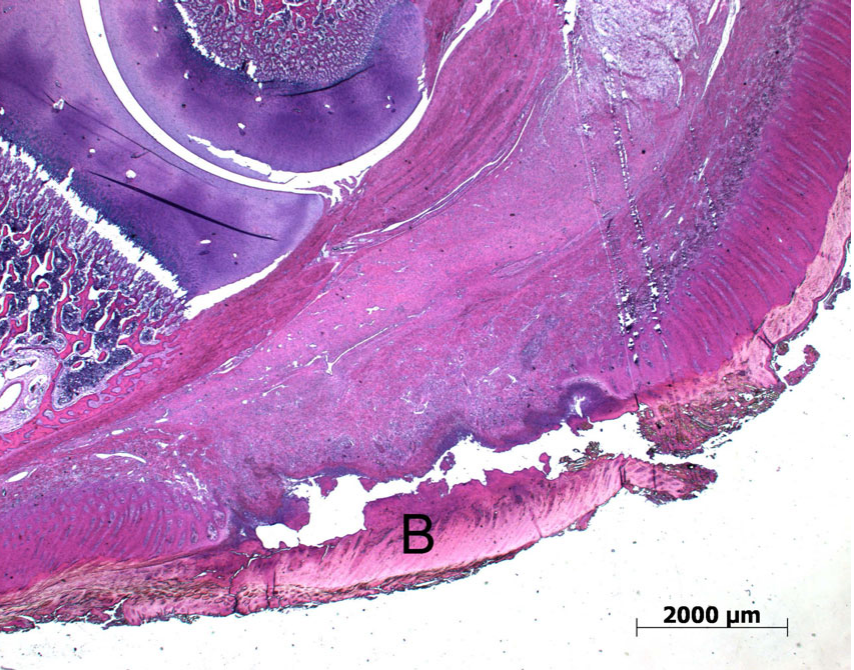
**Histological section of a piglet's heel (toe to the left) with inflammation of the heel and a flap of loose horn tissue (B)**.

**Figure 4:**
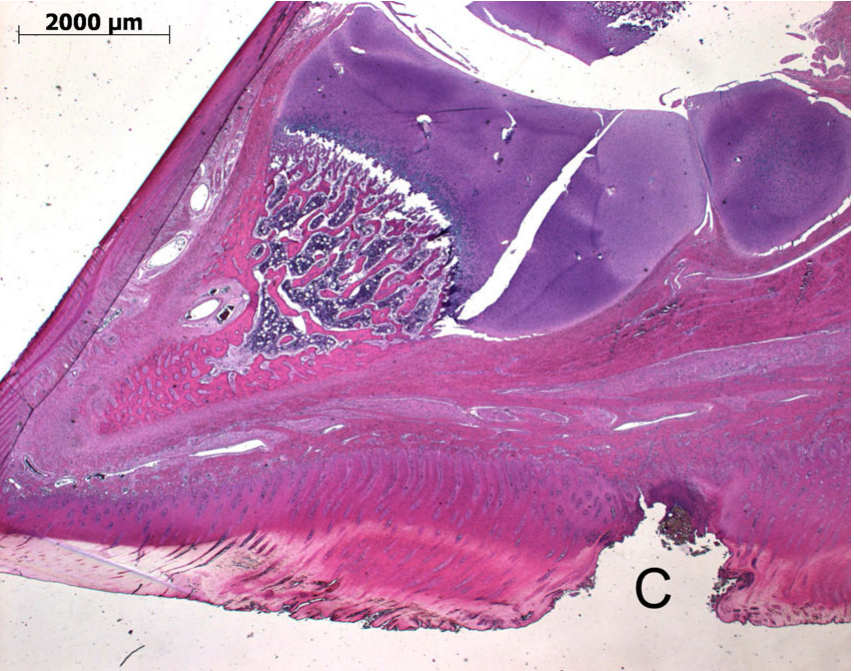
**Histological section of a piglet's claw (toe to the left) with focal pododermatitis (C) of the heel**.

**Figure 5:**
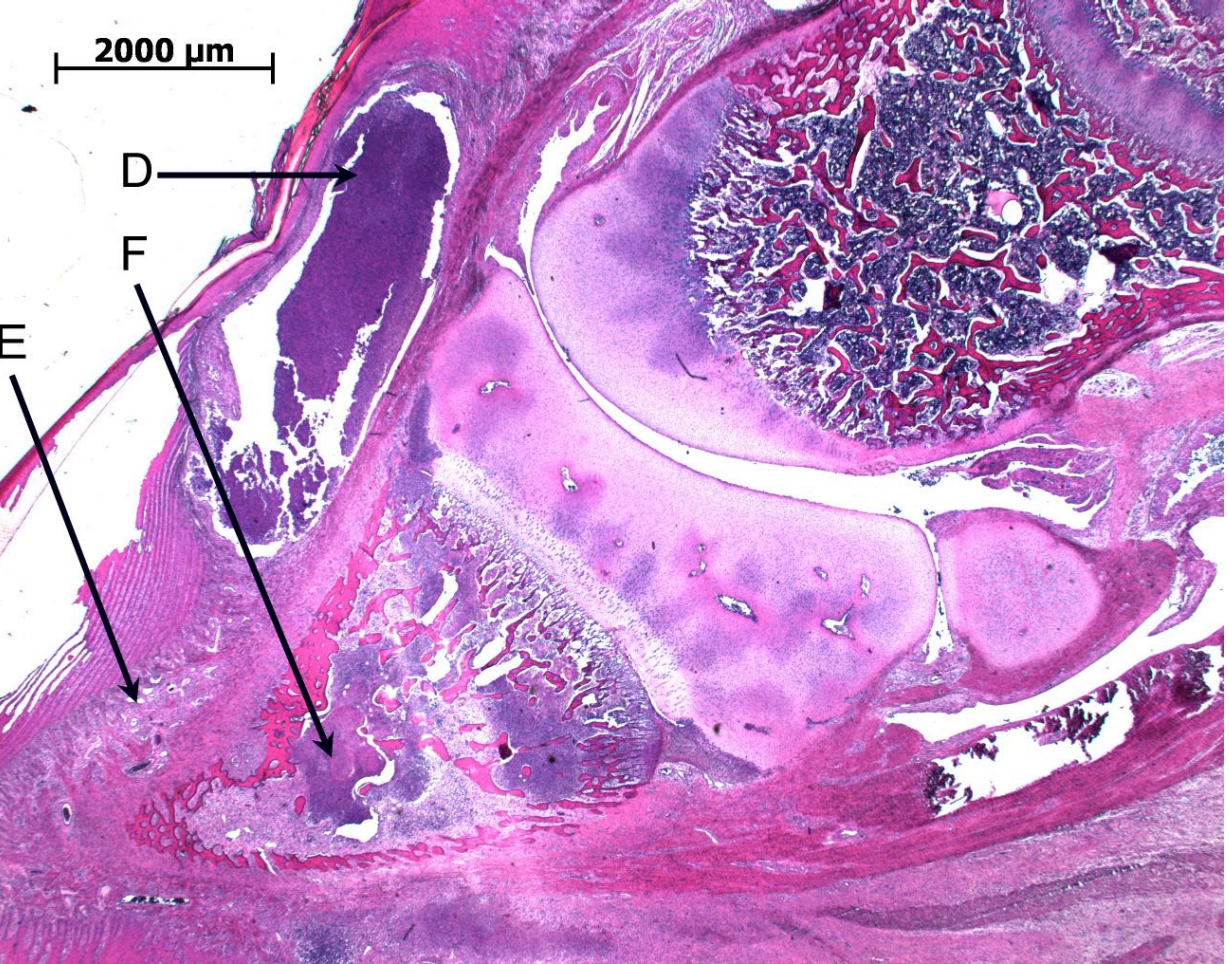
**Histological section of a piglet's claw (toe to the left) with an abscess (D) inflammatory infiltrates (E) and osteomyelitis (F)**.

Skin abrasions were mainly without secondary infection (Figure 2). However, the pathology associated with the foot lesions was more severe. Pathological alterations included necrosis in the horn layers with inflammation of the heel and formation of a flap of horn (B) (Figure 3). Ulceration of the heel horn with focal pododermatitis also occurred (Figure 4). In the most severe examples large abscesses were present, between the coronary band and the wall horn (Figure 5D). In this case inflammatory infiltrates extended all the way down the wall to the tip of the toe (Figure 5E) and there was osteomyelitis of the third phalanx with purulent inflammation and extensive necrosis and dissolution of the bone (Figure 5F).

There was poor correlation between the external appearance of lesions on feet and the extent of inflammation and infection evident at pathological examination. Not all claws that were infected were visibly swollen. The samples selected clinically as examples of unaffected feet and limbs were normal *post-mortem*.

#### Population attributable fractions

Based on the association between floor type and foot and limb lesions reported in the current study, the prevalence of lesions in the affected population would be reduced by between 68% and 100%, if piglets currently housed indoors were housed outdoors. For all types of foot and limb injury the largest proportion of lesions was attributable to partly slatted pens without bedding because this was the most common floor type (Table 9).

**Table 9:** Population attributable fractions for foot and limb lesions in 2878 preweaning piglets from 88 English farms

	**Sole bruising**	**Sole erosion**	**Skin abrasion**	**Swollen joint/claw**
Outdoors				
Solid with bedding	8.7	5.5	12.3	9.4
Part slatted with bedding	19.5	8.5	15.9	9.2
Part slatted some bedding	19.6	19.1	18.0	23.6
Part slatted no bedding	35.2	33.8	29.9	35.4
Fully slatted	14.3	1.4	13.0	21.4
**Total reduction**	97.3	68.3	89.1	100

## Discussion

The current study is, to the authors' knowledge, the largest cross sectional study of the prevalence of foot and limb lesions in preweaning piglets to date. The study farms were approximately 5% of the target population and were a good representation of the English pig farm population in herd size, geographical location and ratio of indoor to outdoor farms from DEFRA 2003 statistics [12]. There may have been a bias towards herds with higher health and welfare standards because the sampling frame was membership of an assurance scheme and compliance in this study was voluntary. As such, prevalence of lesions may be underestimated, which only highlights further the high prevalence of injury in piglets on commercial farms in England. Associations between exposures and disease are unlikely to be affected by self-selection bias.

This study is the first to examine piglets housed outdoors. Soil, with a deep covering of straw, provides a soft, non abrasive surface that was associated with a very low prevalence of foot and limb lesions in piglets. None of the indoor floor types currently used in commercial pig farms in Britain were similar to the outdoor environment and the prevalence of foot and limb lesions was very much higher on all these floors, consequently none can be considered ideal for piglets. Current research of pain in non human animals indicates that injures to the epidermis and deeper tissues and associated inflammation and infection, such as those observed in indoor housed piglets, are likely to be painful [16] and therefore associated with a welfare cost. While it is not possible to measure the pain an animal might be experiencing, the welfare cost associated with foot and limb lesions has been illustrated by reduced activity and play in affected piglets [2]. This is not to say that the welfare of piglets overall can be considered to be better outdoors, such an assessment is beyond the scope of this article and housing pigs outdoors is not a viable option in all localities.

The type of injury that occurred indoors was associated with the floor construction and condition. Slatted floors were associated with an increased risk of sole bruising, perhaps because of the lack of bedding and the increased pressure on the weight bearing areas of the foot resulting from the voids in the floor. The voids might also cause a particular problem when the piglets' claws were small enough to enter the void and pressure from the edge of the slat might bruise the sole.

Areas of solid concrete without bedding (occurring in partly slatted pens) were probably associated with sole erosion because concrete was abrasive. However, even a sparse covering of bedding over concrete floors gave piglets some protection against this lesion. The reduced risk of sole erosion associated with a wet floor in the sow lying area might have occurred because this deterred piglets from this area and they spent more time in dry, possibly bedded creep areas [3]. Alternatively, a wet floor might be a proxy for a floor construction variable that was not measured. There was a trend for a higher prevalence and larger size of sole erosion on the hind feet. This might occur because the piglets push forward with their hind limbs when suckling from the sow [6].

The risk of skin abrasion also increased when the floor was worn and rough and, in contrast to sole erosion, with small amounts of bedding, as reported in previous studies [1,3,5]. It is possible that bedding does not protect against skin abrasions, as it does sole erosions, because skin abrasions occur predominantly while the piglets are scrabbling on their knees to feed, therefore small amounts of bedding quickly get pushed aside, and may even get forced into the skin. Fully slatted floors were associated with a reduced risk of skin abrasion in piglets aged 1-week old or less compared with solid concrete floors. It is likely this occurred because metal or plastic slats are less abrasive than solid concrete. It is possibly that there were also less skin abrasions on partly slatted floors without bedding because these floors had a greater proportion slatted than pens in which bedding was provided. The effect of floor type might only have been significant in young piglets because older piglets, where the abrasions had healed, were misclassified as unaffected.

The results from this study do not support the hypothesis that slatted floors reduce contact between piglets and pathogens and therefore reduce the risk of infections in the feet and limbs. In contrast, slatted floors were associated with an increased risk of swollen joints and claws. Further research is required to understand whether floor type is causal or whether a correlated herd or management factor explains the association. The increased risk of swollen joints and claws associated with a worn floor surface, and a trend for an increased risk with metal slats compared with plastic, might have occurred because these floors are harder to keep clean, or because these features occurred in older pens which may be associated with generally lower standards of housing and management. It is unclear whether the zero prevalence of foot and limb infections in piglets housed outdoors occurred because there were fewer entry sites for infection e.g. tail and tooth clipping and fewer foot and limb injuries, or because the piglets had less contact with pathogens, it is likely to be a combination of both effects.

Overall the association between injuries that might act as entry sites for infection (skin abrasions or sole erosions) and swollen joints or claws was weak, although statistically significant due to the large sample size. A cross sectional design is not ideal to identify such associations because external lesions might have resolved by the time swollen joints or claws developed. The results from the pathology study indicated that the internal pathological changes were commonly more severe than the degree of external damage suggested and infection could be present without visible swelling. Therefore, the reported prevalence of infection might be an underestimation of the true prevalence. It is also possible that not all entry sites for infection were recorded in the cross sectional dataset. Infection in some feet examined *post-mortem *appeared to derive from damage (necrosis) to the coronary band, possibly caused by pressure on the coronary band from the edge of a slat when the claw is small enough to go into the void. This lesion was not recorded in the cross sectional study therefore the prevalence is unknown. This should be addressed in future studies of foot lesions in piglets.

As previously reported [1,4,5], hairless patches and skin abrasions were more prevalent and larger on the fore limbs. The highest prevalence occurred on the carpal joint, which takes the majority of the weight of the piglet when it kneels to suckle. The high prevalence of sole bruising and skin abrasion in the first week of life which then decreased with age has been reported in several previous studies [2,3,5,7,17]. It is likely that the feet and limbs of newborn piglets are particularly soft and vulnerable and then harden with age. But it is unknown whether piglets protected against injury at this stage would simply develop this damage at a later age.

One of the strengths of the current study is that the impact of the floor on the sow [12] and piglets can be compared (Table 10). Lactating sows housed outdoors also had a significantly lower prevalence of limb lesions compared with sows housed indoors. Although the prevalence of limb lesions in outdoor housed lactating sows was considerably higher than in outdoor housed piglets. This might indicate that these lesions develop over time even in the softer outdoor environment, or that these sows have been housed indoors previously. One of the advantages of sampling piglets, compared with older pigs, is that they do not usually move housing during the preweaning period and so it is easier to be sure that the injuries are associated with the environment in which they were observed.

**Table 10:** Summary of associations between limb, body and foot lesions and farrowing pen floor type in lactating sows and piglets

	Lactating sows					Piglets		
**Floor type**	Callus	Wound on limb	Bursitis	Capped hock	Body lesion	Skin abrasion	Sole bruise	Sole erosion
Solid with bedding								
Partly slatted with bedding	▲		-		-	-	-	-
Partly slatted with bedding in some areas*	NA	NA	NA	NA	NA	-	▲	▲
Partly slatted with no bedding	▲	-	-	▲	-	▼	▲	▲
Fully slatted	▲	▲	-	▲	-	▼	▲	-
Outdoor housing	▼	▼	▼	▼	▼	▼	▼	▼

▲ = an increase in risk compared with solid concrete floors with bedding

▼ = a decrease in risk compared with solid concrete floors with bedding

- = no significant difference compared with solid concrete floors with bedding

*this category was not applicable to sows restrained within farrowing crates

The potential conflict between the optimal flooring for sows and piglets reported by previous researchers [1] was evident in the current study. Slatted floors increased the prevalence of wounds on the limbs, and possibly bodies, of lactating sows; perhaps because they increase the pressure on weight bearing areas whilst sows are lying and because these floors were not bedded. Conversely, these same slatted floors were associated with a lower prevalence of skin abrasions on the piglets' limbs in their first week of life. However, it is worth noting that if it were practical to provide sows and piglets housed indoors with solid floors with sufficiently deep bedding to protect the pig from the surface of the concrete, then there might be a lower prevalence of lesions in both sows and piglets, as observed in outdoor housed pigs.

## Conclusion

Piglets housed outdoors in huts with deep straw bedding had a very low prevalence of foot and limb lesions. In piglets housed indoors, no one floor type was ideal; slatted floors were associated with an increased risk of sole bruising and swollen joints or claws but were associated with a reduced risk of skin abrasions in young piglets. Partly slatted floors without bedding were associated with an increased risk of sole erosion. When compared with the risks for limb and body lesions in the piglets' mothers, the lactating sows, outdoor housing was again associated with the lowest prevalence of injury, indicating a good physical environment for both sows and litters. However, indoors no floor type was associated with the lowest prevalence of all types in lesions in sows and piglets. It is likely that the optimal indoor floor type for sows and piglets foot and limb injuries would be a solid floor with deep bedding.

## Abbreviations

The following abbreviations were used in this paper; (OR): Odds ratio; (CI): Confidence interval; (IQR): Interquartile range; (SE): Standard error; (Var.): Variance; (ABP): Assured British Pigs; (DEFRA): Department for Environment, Food and Rural Affairs.

## Competing interests

The authors declare that they have no competing interests.

## Authors' contributions

ALK: participated in the study design, data collection and data management, carried out the data analysis and drafted the manuscript. CEG: provided advice and assistance with data analysis. OP: Carried out pathological and histological examination of foot and limb lesions and made the photographs. LEG: Conceived the project, designed the study, oversaw project management and supervised statistical analysis and assisted with preparation of the manuscript. All authors have read and contributed to the final draft of the manuscript.
